# Oligochitosan fortifies antioxidative and photosynthetic metabolism and enhances secondary metabolite accumulation in arsenic-stressed peppermint

**DOI:** 10.3389/fpls.2022.987746

**Published:** 2022-10-11

**Authors:** Bilal Ahmad, Tariq Ahmad Dar, M. Masroor A. Khan, Ajaz Ahmad, Jörg Rinklebe, Yinglong Chen, Parvaiz Ahmad

**Affiliations:** ^1^Department of Botany, Aligarh Muslim University, Aligarh, India; ^2^Department of Botany, Government Degree College for Women, University of Kashmir, Pulwama, India; ^3^Department of Clinical Pharmacy, College of Pharmacy, King Saud University, Riyadh, Saudi Arabia; ^4^Laboratory of Soil- and Groundwater-Management, School of Architecture and Civil Engineering, Institute of Soil Engineering, Waste- and Water Science, University of Wuppertal, Wuppertal, Germany; ^5^International Research Centre of Nanotechnology for Himalayan Sustainability (IRCNHS), Shoolini University, Solan, India; ^6^The UWA Institute of Agriculture, and School of Agriculture and Environment, The University of Western Australia, Perth, WA, Australia; ^7^Department of Botany, Jammu and Kashmir, India

**Keywords:** chitosan, arsenic (As) toxicity, peppermint, enzymatic (ascorbate peroxidase, superoxide dismutase) antioxidants, non-enzymatic (glutathione) antioxidants

## Abstract

The current study was designed to investigate whether application of irradiated chitosan (ICn), a recently established plant growth promoter, can prove effective in alleviating arsenic (As) stress in peppermint, a medicinally important plant. This study investigated how foliar application of ICn alleviated As toxicity in peppermint (*Mentha piperita* L.). Peppermint plants were treated with ICn (80 mg L^−1^) alone or in combination with As (10, 20, or 40 mg kg^−1^ of soil, as Na_2_HAsO_4_·7H_2_O) 40 days after transplantation (DAT), and effects on the growth, photosynthesis, and antioxidants were assessed at 150 DAT as stress severely decreases plant growth, affects photosynthesis, and alters enzymatic (ascorbate peroxidase, superoxide dismutase) and non-enzymatic (glutathione) antioxidants. When applied at 40 mg kg^−1^, ICn significantly decreased the content of essential oil (EO) and total phenols in peppermint by 13.8 and 16.0%, respectively, and decreased phenylalanine ammonia lyase (PAL) and deoxy-D-xylulose-5-phosphate reductoisomerase (DXR) activities by 12.8 and 14.6%, respectively. Application of ICn mitigated the disadvantageous effects caused by As toxicity in peppermint by enhancing activities of antioxidative enzymes and photosynthesis and increased accretion of secondary metabolism products (EOs and phenols). An enhancement of total phenols (increased by 17.3%) and EOs (36.4%) is endorsed to ICn-stimulated enhancement in the activities of PAL and DXR (65.9 and 28.9%, respectively) in comparison to the control. To conclude, this study demonstrated that foliar application of ICn (80 mgL^−1^) effectively promoted the growth and physiology of peppermint and eliminated As-induced toxicity to achieve high production of EO-containing crops grown in metal-contaminated soils.

## Introduction

Arsenic (As), a ubiquitous potentially noxious metalloid, exists as inorganic or organic forms, with arsenate (As^V^) and arsenite (As^III^) as the primary inorganic forms, in the environment (Rahaman et al., 2021; Rehman et al., 2021). It is becoming a severe disquiet due to its toxicity in humans, animals, and plants and hence regarded as a priority contaminant (Li et al., 2018; Rahaman et al., 2021). Prolonged exposure to As leads to a variety of lethal diseases in humans, notably cardiovascular and neurological disorders, and various types of skin cancers (Kumar et al., 2015; Li et al., 2017; Jia et al., 2020; Rehman et al., 2021). Millions of people worldwide fall victims to As contamination mainly through consumption of food and drinking water contaminated with As (Zhao et al., 2013; Rahaman et al., 2021). As enters into agricultural systems through mining, application of As-based pesticides, consumption of or irrigation with As-polluted groundwater, employing municipal solid wastes as fertilizers, and natural geochemical processes (Wilson and Pyatt, 2007; Nabi et al., 2021; Rehman et al., 2021; Zhang et al., 2021). The recommended safe concentration of As in drinking water is 10 μg L^−1^; nevertheless, groundwater As has attained a level of about 3,200 μg L^−1^ in the northern parts of Indian subcontinent (McCarty et al., 2011). In addition, the concentration of As in agricultural soils irrigated with As-polluted groundwater is distressingly amplifying, affecting the productivity of plants severely (Flora et al., 2007; Nabi et al., 2021; Zhang et al., 2021). As contamination disrupts soil quality, inhibits root growth, and impairs primary (photosynthesis and respiration) and secondary metabolic processes (Abbas et al., 2018; Kumari et al., 2018; Suriyagoda et al., 2018; Nabi et al., 2021; Zhang et al., 2021).

Employing natural polysaccharides in oligomeric forms, in view of their plant growth-promoting effect, is a proficient agricultural practice, and considerable success of this practice is accredited to the exceptional biological characteristics of the oligomers (biodegradability, biocompatibility, and non-toxicity) (Abad et al., 2016; Ahmad et al., 2017, 2019a,b; Shabbir et al., 2017; Naeem et al., 2020). Moreover, polysaccharides processed through irradiation are flourishing as potent abiotic stress alleviators (Singh et al., 2017; Naeem et al., 2020). Among the different polysaccharides employed in oligomeric forms, chitosan (obtained through incomplete chitin de-acetylation) fulfills several functions in agriculture, particularly coating of eatables, plant growth promoter, and antibacterial activities (Kumar, 2000; Jeon et al., 2001; Katiyar et al., 2014). Chitosan has emerged as an encouraging substitute in the agricultural field in view of its outstanding properties, notably non-antigenicity, antifungal and antimicrobial properties, and biodegradability (Kumar, 2000; Kurita, 2006; Katiyar et al., 2014; Ahmad et al., 2017). Irradiated chitosan (ICn) obtained through radiolytic degradation of chitosan has been reported to attain comprehensive plant growth-promoting activity as compared to un-irradiated forms (Dar et al., 2015; Ahmad et al., 2017; Ahmed et al., 2020). ICn applied through foliage improves the activity of different carbon and nitrogen metabolism enzymes in various medicinal plants, leading to increased plant growth, photosynthesis, nutrient assimilation, and secondary metabolite production (Dar et al., 2015; Ahmad et al., 2017; Jaleel et al., 2017; Ahmed et al., 2020).

Despite the advancement in modern pharmacology, application of medicinal and aromatic plants (MAPs) and their products as alternative curative medicines has increased significantly, with advantages of being cheap, easily affordable, and hazardless in contrast to the contemporary synthetic pharmaceutics (Khan and Smillie, 2012). Among the most vital MAPs, peppermint (*Mentha piperita* L.) encompasses colossal pharmaceutical implication in view of its anti-convulsive, antiseptic, and anesthetic properties (Szymczycha-Madeja et al., 2013; Mahendran and Rahman, 2020). Many peppermint properties, notably anti-inflammatory, antiviral, antimicrobial, antiulcer, cytoprotective, hepatoprotective and antispasmodic values, add to the therapeutic significance of the plant (Shah and Mello, 2004; Pytlakowska et al., 2012). Moreover, it also contains other therapeutic properties, including vasodilation, gastritis, flatulence, colitis, stimulation of bile production, and increased endocrine secretions (Uribe et al., 2016; Mahendran and Rahman, 2020). Peppermint essential oil (EO) is dominated by volatile isoprenoids including menthol, which constitutes one of the most desired flavor being part of food, flavorings, cosmetics, and pharmaceutics across the globe (Uribe et al., 2016; Mahendran and Rahman, 2020). Medicinal characteristics are predominantly accredited to the monoterpenes in the EO, among which menthol and menthyl acetate produce a cooling sensation (Mimica-Dukic and Bozin, 2008; Pharmacopoeia, 2008; Pytlakowska et al., 2012). In addition to being used in food industries, polyphenols have rich antioxidant properties and thus add to the medicinal significance of the plant [38, 39]. Secondary metabolites, particularly phenols, serve as potent abiotic stress alleviators in plants, in addition to many other chemo-ecological roles (Márquez-García et al., 2012; Kisa et al., 2016; Naikoo et al., 2019; Kaya et al., 2020). India holds around 60% share in the total world mint oil production (Pérez et al., 2014; Kumari et al., 2018). The content of EO in peppermint seldom exceeds 1%, and the metal/metalloid pollution of the agricultural soils further curtails the crop yield, adding to the distress of farmers by callously affecting their economy. Moreover, the consumption of As-contaminated crop can lead to severe health hazards. Therefore, this study aimed to document whether oligochitosan can alleviate the detrimental effects of As on peppermint and improve its performance. The outcomes of this study will gain insights into the response of peppermint to As stress to enhance our understanding of As-induced toxicity on plant performance and secondary metabolites.

## Materials and methods

The experiment was executed in the screen house (Department of Botany), Aligarh Muslim University, Aligarh (78°51′ E, 27°52′ N, 187.45 m altitude), under natural conditions. The mean temperature for the experimental period was record as follows: 7.6°C (December), 7.2°C (January), 11.5°C (February), 19.1°C (March), 24.5°C (April), and 27.1°C (May). Average precipitation during the experiment was 4.6–15.7 mm, and the relative humidity was 38%−77%. Plastic pots (40 cm diameter ×45 cm depth) were each filled with 8.5 kg of farm soil mixed with organic manure (5:1).

Vigorous rhizomes of peppermint (*Mentha piperita* L.) were used for plant propagation. The rhizomes were surface-sterilized for 5 min using a HgCl_2_ solution (0.02%), followed by repeated shaking and rigorous washing with de-ionized (DI) water prior to transplantation. A simple randomized design was used for the current study. Each pot was filled with 8.5 kg of a homogeneous mixture of soil and organic manure prior to transplantation. Properties of the mixed soil were tested at the Indian agricultural Research Institute (IARI), New Delhi: sandy loam texture, E.C. (1:2) 0.37 m mhos cm^−1^, pH (1:2) 8.03, available N, P, and K; 94.2, 9.5, and 136.2 mg kg^−1^ of soil, respectively, and no As was detected in the soil. A uniform basal split dose of N, P, and K (30 + 30, 30 + 30, and 30 + 30) mg kg^−1^ soil, respectively, was applied in the form of urea, single superphosphate, and muriate of potash, respectively. The second split dose was applied to the soil after 1 month.

In the screening experiment, three arsenic (As) concentrations (10, 20, and 40 mg kg^−1^ of soil, in the form of Na_2_HAsO_4_·7H_2_O) were chosen based on the results from an early screening experiment for As toxicity testing of various As concentrations applied to soil 40 days after transplantation (DAT). Irradiated chitosan (ICn), a natural nitrogen-containing non-toxic and biodegradable carbohydrate polymer processed through irradiation, was used in this study in view of its exceptional elicitor activity attained due to irradiation [18, 19, 27]. ICn concentration (80 mg L^−1^) was selected from another screening experiment conducted on the same plant (Ahmad et al., 2017) and applied alone or in combination with the three As concentrations. ICn was dissolved in 0.03% acetic acid, and thereafter, the desired concentration (80 mg L^−1^) was prepared using DI water. ICn was applied using a hand sprayer for seven times started on 50 DAT and every 7 days thereafter. An equal amount of DI water was sprayed on the control plants. Therefore, the experiment included two factors (three As treatments and two ICn treatments) and five replicates (pots), with three plants per pot. Weeding and watering of the plants were carried out, as and when required.

### Irradiation of chitosan

A gamma radiation chamber (Cobalt-60, GC-5000, BRIT, Mumbai, India) was employed for the irradiation of chitosan, which was originally procured from an Indian marine chemicals company. Irradiation of the chitosan samples was carried out at a total dose of 250 kGy using Co-60.

### Determination of growth attributes

Determination of growth attributes was accomplished at 150 DAT; one plant from every replicate of each pot (replicate) was first uprooted with care, followed by thorough and careful cleaning. Plant height and fresh weight (FW) of each plant were measured. Plants were oven-dried at 80°C for 24 h for the determination of dry weight (DW).

### Estimation of total chlorophyll content, net photosynthetic rate, and stomatal conductance

The method of Lichtenthaler and Buschmann (2001) was adopted for the estimation of the content of total chlorophyll in the fresh leaves. The interveinal leaf tissue was grinded with acetone (100%) using a mortar and pestle. After centrifugation, the optical density (OD) of the pigment solution at two different wavelengths (662 and 645 nm) was recorded using a spectrophotometer (Shimadzu UV-1700, Tokyo, Japan). These ODs were used for the determination of the contents of chlorophyll a and chlorophyll b.

The net photosynthetic rate (P_N_) and stomatal conductance (g_s_) were estimated from the first fully expanded leaves on bright cloudless days at 1100 h by means of an infrared gas analyzer (IRGA, LI-COR 6400 Portable Photosynthesis System, Nebraska, USA).

### Chlorophyll fluorescence estimation

Chlorophyll fluorescence (Fv/Fm) was estimated using a saturation-pulse fluorometer PAM-2000 (Walz, Effeltrich, Germany). All measurements were carried out in the diurnal time on the first pair of unifoliolate, fully expanded leaves. The upper surface of leaves was clipped to estimate the chlorophyll fluorescence.

### Estimation of enzyme activities

#### Estimation of ribulose bisphosphate carboxylase oxygenase and phenylalanine ammonia lyase activity

Homogenization of peppermint leaves (100 mg) with liquid nitrogen in a mortar and pestle containing a buffer solution of pH 7.8 (1 mM EDTA and 50 mM potassium phosphate) and 1% polyvinyl pyrrolidone (PVP) was carried out. Thereafter, the homogenized leaf material was filtrated through a nylon filter (0.20 mm) inside a centrifuge tube. The leaf tissue extract was centrifuged at 12,000 g for 40 min at 4°C. The supernatant was preserved at −20°C for the assessment of RuBisCo activity by using a spectrophotometric method of Usuda (1985).

The method of Beaudoin-Eagan and Thorpe (1985) was for estimating the PAL activity by assessing the quantity of trans-cinnamic acid formed at 290 nm. A measure of 100 mL of enzyme extract, 900 mL L-phenylalanine (6 mM), and 500 mM Tris–HCl buffer solution (pH 8) constituted the reaction mixture. The reaction mixture was placed in a water bath for 70 min at 37°C. The reaction was terminated by adding 50 mL of 5 N HCl. Trans-cinnamic acid (1 mg mL^−1^) was used as a standard, and the activity of PAL was expressed as mmol trans-cinnamic acid min^−1^ mg^−1^ protein.

#### Estimation of carbonic anhydrase and nitrate reductase activities

NR activity (EC 1.7.1.1) was determined using the method (intact tissue assay) devised by Jaworski (1971). Likewise, the activity of CA was calculated from the fresh leaves, following the method of Dwivedi and Randhawa (1974).

### Estimation of deoxy-D-xylulose-5-phosphate reductoisomerase activity

DXR activity in the peppermint leaves was estimated using the spectrophotometric method originally adopted by Ramak et al. (2013). The change in absorption due to oxidation of NADPH to NADP^+^ was observed at 340 nm, and the activity of DXR was determined.

### Determination of total phenol content

The determination of total phenols was carried out by colorimetry at a wavelength of 760 nm (Singleton and Rossi, 1965). Plant extract from each treatment (0.5 mL), as well as gallic acid (used as standard phenolic compound), was mixed with an aqueous Na_2_CO_3_ (1 M, 1 mL) and Folin–Ciocalteu reagent (0.5 mL, diluted with 8 mL distilled water). Estimation of total phenols was carried out at a wavelength of 760 nm using colorimetry. The total phenol content was then estimated as mg gallic acid equivalent g^−1^ plant DW.

### Estimation of proline content

The method devised by Bates et al. (1973) was employed for the estimation of the leaf proline content. Fresh leaf samples were homogenized after adding sulfosalicylic acid. Glacial acetic acid and ninhydrin solutions were added to the leaf extract in equal proportions. Thereafter, toluene (5 mL) was added to the samples after heating at 100°C. Using a spectrophotometer, the toluene layer absorbance was noted at 520 nm.

### Assay of antioxidant enzymes

Fresh leaves (200 mg) were homogenized in an ice-cold mortar and pestle using an extraction buffer [100 mM potassium phosphate buffer (pH 7.0) added with 0.05% Triton X-100 (v/v) and 1% polyvinyl pyrrolidone (w/v)]. The homogenate was centrifuged at 15,000 × g for 20 min at 4°C. The supernatant obtained was assayed for estimation of superoxide dismutase (SOD). SOD activity was estimated by assessing the inhibition of nitro blue tetrazolium (NBT) photochemical reduction, following the procedure devised by Giannopolitis and Ries (1977) and Beyer and Fridovich (1987). Likewise, peroxidase activity was estimated as per the procedure devised by Chance and Maehly (1955). The activity of GR was estimated by assessing the GSH-dependent oxidation of NADPH at 340 nm, following the method described by Foyer and Halliwell (1976). The estimation of APX activity was carried out by monitoring the decrease in the absorbance of ascorbate at 290 nm, following the method of Nakano and Asada (1981).

### Estimation of TBARS

The thiobarbituric acid-reactive substance (TBARS) content was estimated for the determination of lipid peroxidation in leaves as per the procedure devised by Cakmak and Horst (1991). Fresh leaves (500 mg) were chopped and grinded with 5 mL of 0.1% (w/v) trichloroacetic acid (TCA). The mixture was then centrifuged for 5 min at 12,000 × g, and an aliquot of 1 mL of the supernatant was added to 4 mL of 0.5% (w/v) TBA in 20% (w/v) TCA. The samples were then incubated for 30 min at 90 C. The reaction was terminated by placing them in an ice bath. The mixture was centrifuged at 10,000 × g for 5 min, and the absorbance of the supernatant was recorded at 532 nm using a spectrophotometer (Shimadzu UV-1700, Tokyo, Japan). Correction of the values for non-specific turbidity was performed by subtracting the absorbance at 600 nm. The TBARS content was expressed as nanomoles per gram FW.

### Estimation of carbohydrate content

Estimation of the leaf carbohydrate content was accomplished following the procedure devised by Dubois et al. (1956). First, 0.5 mL phenol (5%) was added to 1 mL leaf extract in a test tube which was placed in cool water. After that, 2.5 mL H_2_SO_4_ was added to the test tube. The optical density was then recorded at 490 nm by means of a spectrophotometer (Shimadzu UV-1700, Tokyo, Japan).

### Estimation of arsenic content

The As content in the plant tissues was estimated following the procedure of Rai et al. (2011). The root and shoot tissue samples were first dried, followed by grinding it into fine powder. Thereafter, the samples were digested in HNO_3_ and H_2_O_2_ (3:1) until the solution turned transparent. The digested root and shoot samples were then used for the estimation of the leaf As content. The As content was estimated using an atomic absorption spectrometer (graphite furnace-fitted) (Perkin Elmer; A Analyst 600).

### Isolation and compositional analysis of essential oil

Fresh leaves plucked from each replicate were chopped, mixed, and used for the extraction of essential oil (EO) as per the method of Guenther (1972). The Clevenger apparatus was used for the extraction of EO. EO was then collected, dried (using anhydrous sodium sulfate), and preserved at a temperature of 4°C inside sealed glass vials for the analysis of monoterpenes and individual components by gas chromatography–mass spectroscopy [GC–MS (Shimadzu QP−2010 equipped with thermal desorption (TD 20) system (Rtx-5) with a fused silica capillary column (30 m ×0.25 mm ×0.25 μm]. The temperature schedule of GC–MS was as follows: oven temperature, 270°C; detector temperature, 260°C; column temperature, 250°C (with a hold time of 2 min) at a rate of 5°C min^−1^, and then 260–280°C at 10°C min^−1^ with a finishing hold time of 15 min, using helium (He) gas as a carrier. The injector temperature was 260°C, sample size was 0.2 μL, split ratio was 1:10. The ionization energy of the equipment was 70 eV (EI), with about 40–400 amu mass scan range.

## Results

### Foliar application of chitosan oligomers improves growth in As-stressed peppermint

The promotive effect of ICn on growth parameters is clearly discernible in the current study. Foliar application of ICn significantly increased the values of the FW and DW of the plant under study by 24.4 and 34.5%, while herbage yield and plant height were augmented by 22.9 and 23.7%, respectively, as compared to DI water-sprayed control (*P* < 0.05; Table 1). When As was applied through soil, it severely affected the growth of peppermint. Among the various concentrations of the metalloid, As-40 proved to be the most deleterious (Table 1). Significant reduction of 23.5 and 28.4% in the FW and DW, respectively, was reported over the control (Table 1). Similarly, herbage yield and plant height were significantly decreased by 25.9 and 27.7%, respectively (Table 1). The deleterious effects of differential As concentrations were considerably mitigated by the application of ICn. The FW and DW of the As-40 plants were significantly increased by 11.3 and 16.6%, respectively, due to supplementation of ICn-80 (As-40+ICn-80), while herbage yield and plant height in these plants recorded a significant increase of 15.3 and 22.6%, respectively, as compared to the As-40 treatment.

**Table 1:** Growth parameters of peppermint in As-contaminated soil with and without supplementation of irradiated chitosan (ICn) at 150 DAT (mean of five replicates ± SE).

**Parameters**	**Treatments**							
	**Control (DIW)**	**ICn-80**	**As-10**	**As-20**	**As-40**	**As-10+ICn-80**	**As-20+ ICn-80**	**As-40+ ICn-80**
Fresh weight (g)	78.37 ± 2.66^bc^	97.57 ± 1.66^a^	74.62 ± 2.16^c^	69.71 ± 3.07^cd^	63.45 ± 3.29^d^	84.45 ± 3.13^b^	77.78 ± 2.96^bc^	70.65 ± 2.73^cd^
Dry weight (g)	19.43 ± 0.78^bc^	26.14 ± 0.89^a^	17.95 ± 0.76^c^	16.74 ± 0.68^cd^	15.13 ± 0.55^d^	20.94 ± 0.67^b^	19.04 ± 0.61^bc^	17.64 ± 0.69^c^
Herbage yield (g)	50.13 ± 2.29^bc^	61.65 ± 2.13^a^	46.21 ± 2.27^cd^	43.52 ± 1.98^d^	39.79 ± 2.03^e^	53.56 ± 2.11^b^	48.68 ± 1.87^cd^	45.89 ± 2.38^cd^
Plant Height (cm)	68.3 ± 1.64^c^	84.5 ± 1.72 ^a^	62.2 ± 1.81^d^	56.7 ± 1.74^e^	53.5 ± 1.86^e^	74.4 ± 1.93^b^	69.8 ± 1.89^c^	65.6 ± 2.03^cd^

Means within a row followed by the same letter(s) are not significantly different according to DMRT (*p* ≤ 0.05).

### ICn applied through foliage ameliorates photosynthesis in peppermint under As stress

**Figure 1:**
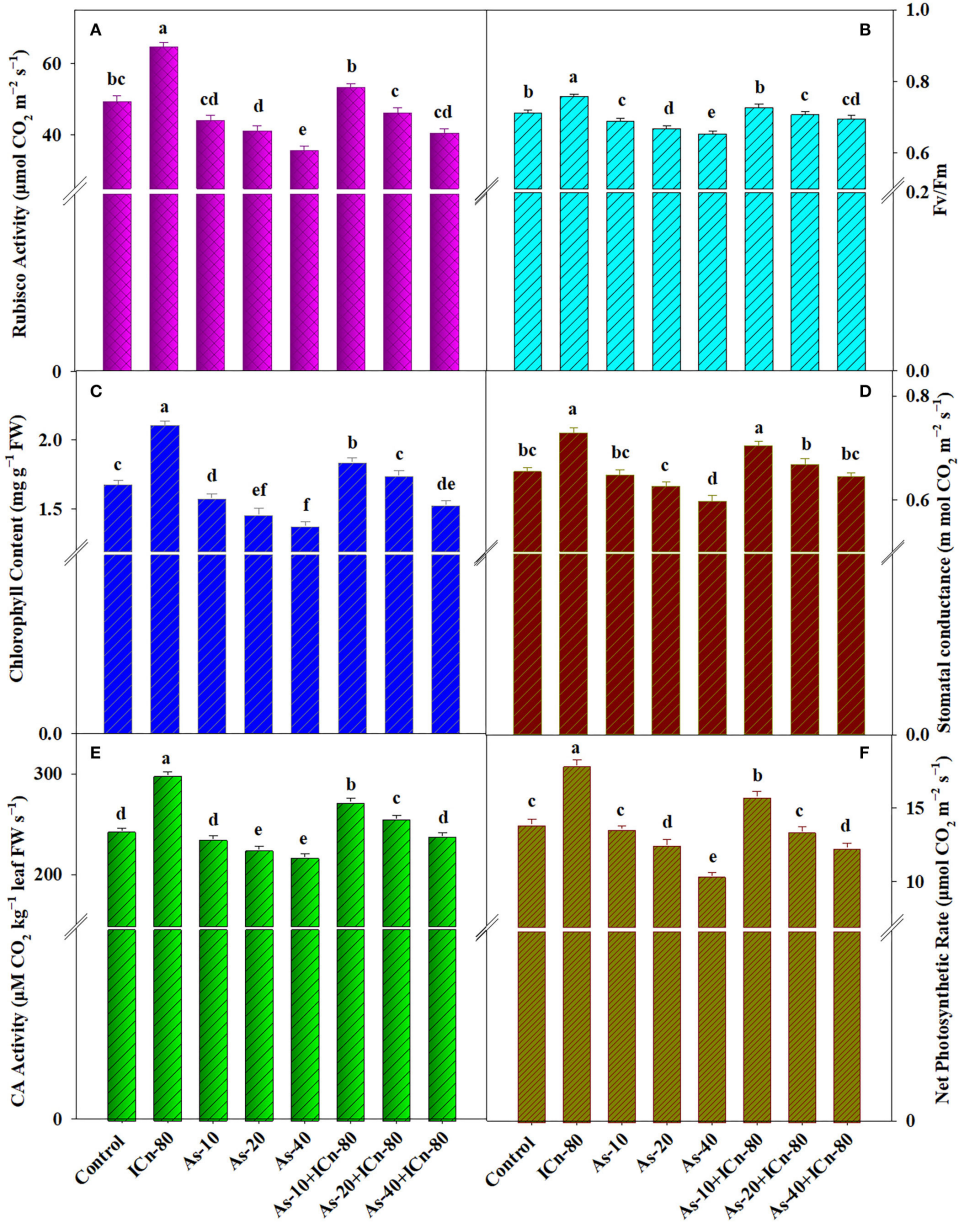
RuBisCo activity **(A)**, chlorophyll fluorescence (Fv/Fm) **(B)**, chlorophyll content **(C)**, stomatal conductance **(D)**, carbonic anhydrase activity **(E)**, net photosynthetic rate **(F)** of peppermint (*Mentha piperita* L.) at 150 days after transplanting. Plants were grown with/without As stress (10, 20, or 40 mg kg^−1^ of soil, as Na_2_HAsO_4_·7H_2_O) and treated with ICn (80 mg L^−1^). Data are presented as treatment mean ± SE (*n* = 5). Means within a column followed by the same letter are not significantly different (*P* < 0.05).

The detrimental effects of As on different photosynthetic attributes of peppermint are clearly evident in the literature. All the concentrations of As (As-10, As-20, and As-40) reduced the growth parameters as compared to the control; nevertheless, a significant decrease was observed in case of As-40-treated plants (*P* < 0.05; Figure 1). RuBisCo and CA activities were significantly decreased by 38.4 and 11.9%, respectively. Similarly, this treatment significantly decreased the total chlorophyll content and stomatal conductance by 21.7 and 9.5%, respectively (Figure 1). Moreover, a significant decrease of 33.6 and 8.9% in NPR and Fv/Fm, respectively, was witnessed compared to the control (Figure 1). Leaf-applied chito-oligosaccharides influenced the photosynthetic attributes positively. Compared to the control, a significant increase of 30.9 and 22.7% in the activities of RuBisCo and CA in ICn-80 was observed (Figure 1). This treatment increased the content of total chlorophyll and stomatal conductance (*g*_*s*_) significantly by 25.6 and 11.5%, respectively, over the control (Figure 1). Similarly, a significant increase of 28.7 and 11.6% in NPR and Fv/Fm was recorded (Figure 1). The deleterious effects of As on the photosynthetic parameters were effectively alleviated by leaf-applied ICn. Foliar application of 80 mg L^−1^ ICn significantly mitigated the negative effects of As-10 and As-20, and the values obtained were either at par or greater than the control. ICn-80 sprayed on As-40-treated plants recorded a significant increase of 22.1 and 9.5% in RuBisCo and CA activities, respectively, in comparison to As-40 treatment. Likewise, the chlorophyll content was significantly improved by 10.9%, while stomatal conductance displayed an improvement of 7.9% compared to the As-40 treatment. Similarly, a significant enhancement of 18.3 and 6.4% in NPR and Fv/Fm, respectively, was reported in As-40 treatment after supplementation with 80 mg L^−1^ ICn (Figure 1).

### ICn fortifies oxidative metabolism in peppermint exposed to As stress

**Figure 2:**
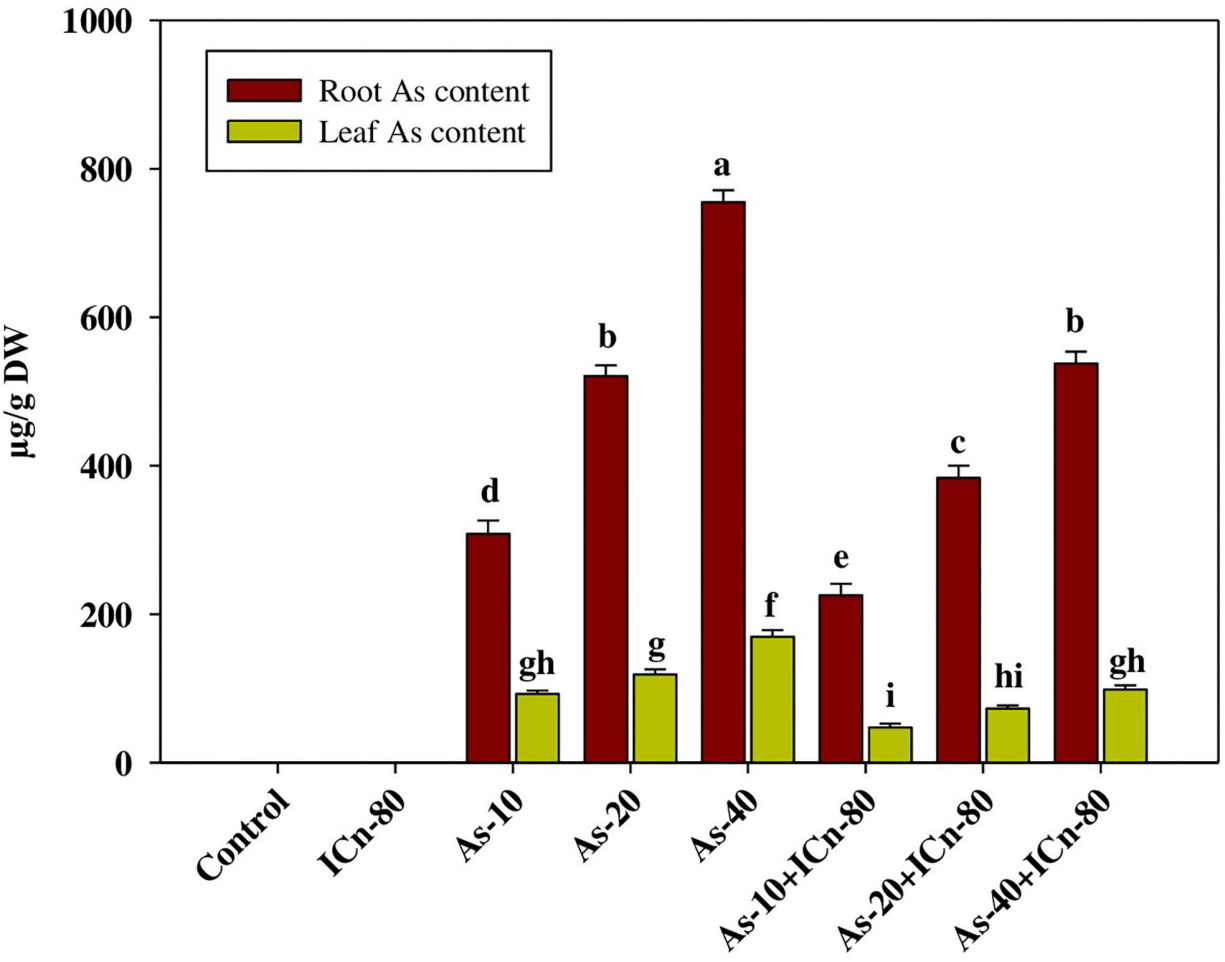
Leaf and root arsenic accumulation of peppermint (*Mentha piperita* L.) at 150 days after transplanting with/without As stress (10, 20, or 40 mg kg^−1^ of soil, as Na_2_HAsO_4_·7H_2_O) and treated with ICn (80 mg L^−1^). Data are presented as treatment mean ± SE (*n* = 5). Bars with the same letters are not significantly dissimilar (*P* < 0.0).

**Figure 3:**
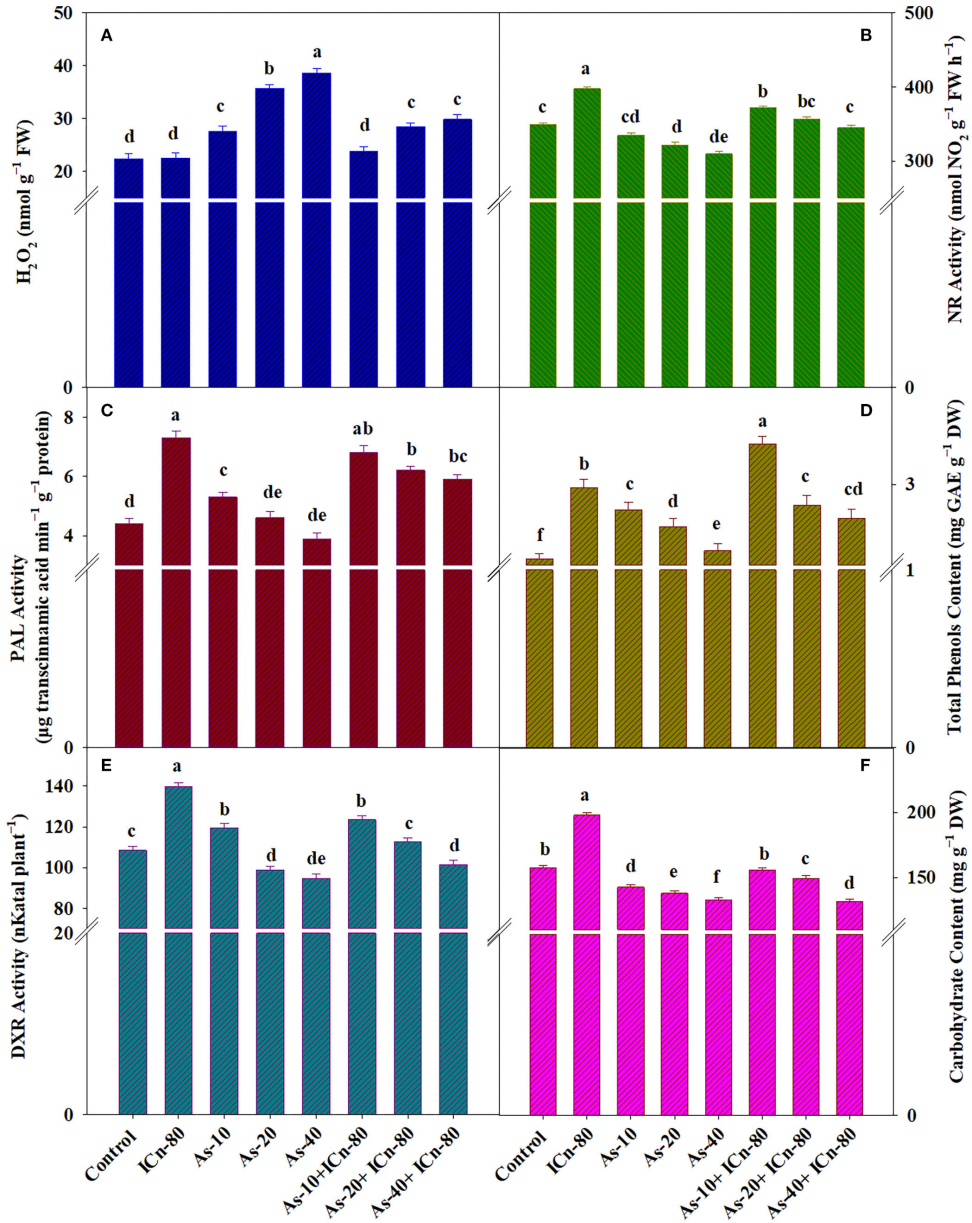
H_2_O_2_ content **(A)**, nitrate reductase activity **(B)**, phenylalanine ammonia lyase (PAL) activity **(C)**, total phenol content **(D)**, deoxy-D-xylulose-5-phosphate reductoisomerase (DXR) activity **(E)**, and carbohydrate content **(F)** of peppermint (*Mentha piperita* L.) at 150 days after transplanting with/without As stress (10, 20, or 40 mg kg^−1^ of soil, as Na_2_HAsO_4_·7H_2_O) and treated with ICn (80 mg L^−1^). Data are presented as treatment mean ± SE (*n* = 5). Bars with the same letters are not significantly dissimilar (*P* < 0.0).

SOD, POX, and APX activities were enhanced significantly in As-treated peppermint (*P* < 0.05; Table 2). Maximum values of SOD, POX, and APX activities were reported in the As-40 treatment. This treatment recorded a significant enhancement of 94.2, 54.5, and 49.2%, respectively, over the control (Table 2). Supplementation of ICn significantly decreased the activities of SOD, POX, and APX enzymes in As-40 plants by 17.4, 12.7, and 9.9%, respectively, as compared to the un-supplemented As-40 treatment. A maximum increase of 54.5% in TBARS over the control was reported in the As-40-treated plants. TBARSs in the metalloid-treated plants after ICn application were observed to decrease, demonstrating the mitigation of oxidative stress. A significant diminution of 19.5% in the TBARS content as compared to As-40 was reported for the As-40+ICn-80 treatment (Table 2). A significant increase of 54.1% was observed in ICn-80 treatment, while the increase was 77.1% in the As-40+ICn-80 treatment over the control (Table 2). GR activity in the As-treated plants was considerably higher, and the highest enhancement was observed in As-stressed plants supplemented with ICn-80 as compared to the control (Table 2). An increase of 92.3% in the GR activity was observed in As-40+ICn-80 as compared to the DI water-sprayed control (Figure 2). Similar to TBARSs, the H_2_O_2_ content was enhanced significantly in peppermint exposed to As toxicity. Maximum accumulation of H_2_O_2_ was reported in As-40 treatment. This treatment exhibited a significant increase of 72.3% over the control plants. ICn proved effective in scavenging the ROS as is evident from the results (Figure 3A). As-40 treatment unveiled a decrease of 29.4% in the H_2_O_2_ content after supplementation with ICn-80.

**Table 2:** Superoxide dismutase (SOD) activity, TBARS, peroxide (POX) activity, glutathione reductase (GR) activity, ascorbate peroxidase (APX) activity, and proline content of peppermint (*Mentha piperita* L.) at 150 DAP.

**Parameters**	**Treatments**							
	**Control (DIW)**	**ICn-80**	**As-10**	**As-20**	**As-40**	**As-10+ICn-80**	**As-20+ ICn-80**	**As-40+ ICn-80**
SOD activity (units mg^−1^ protein)	5.42 ± 0.24^e^	6.21 ± 0.26^d^	7.48 ± 0.37^c^	9.07 ± 0.31^b^	10.53 ± 0.29^a^	6.85 ± 0.25^cd^	7.37 ± 0.32^c^	8.97 ± 0.31^b^
TBARS content (nmol g^−1^ FW)	11.20 ± 0.31^d^	9.40 ± 0.35^e^	13.30 ± 0.26^c^	15.9 ± 0.32^b^	17.30 ± 0.29^a^	11.80 ± 0.38^d^	13.30 ± 0.33^c^	16.10 ± 0.28^b^
POX activity (units mg^−1^protein)	18.30 ± 0.65^f^	19.40 ± 0.62^e^	24.30 ± 0.68^c^	27.90 ± 0.74^b^	31.80 ± 0.73^a^	21.40 ± 0.71^d^	25.10 ± 0.66^c^	28.20 ± 0.79^b^
GR Activity (units mg^−1^ protein)	1.67 ± 0.075^g^	1.82 ± 0.042^f^	2.36 ± 0.061^d^	2.91 ± 0.066^b^	3.25 ± 0.063^a^	2.12 ± 0.052^e^	2.65 ± 0.081^c^	3.08 ± 0.069^ab^
APX activity (units mg^−1^ protein)	4.51 ± 0.14^f^	4.82 ± 0.13^ef^	5.43 ± 0.16^de^	6.07 ± 0.153^bc^	6.73 ± 0.18^a^	5.03 ± 0.16^ef^	5.68 ± 0.13^cd^	6.12 ± 0.17^b^
Proline (mg g^−1^ FW)	4.80 ± 0.59^d^	7.40 ± 0.62^ab^	5.30 ± 0.49^cd^	5.90 ± 0.54^bcd^	6.40 ± 0.51^bc^	7.40 ± 0.46^ab^	8.10 ± 0.51^a^	8.50 ± 0.55^a^

Plants were grown with/without As stress and treated with ICn. Data are presented as treatment mean ± SE (*n* = 5) (mean of five replicates ± SE). Means within a row followed by the same letter(s) are not significantly different according to DMRT (*p* ≤ 0.05).

### ICn reduces As uptake and root-to-shoot translocation of As in peppermint

ICn applied to leaves significantly decreased As accumulation in the roots and leaves of peppermint. A significant decrease of 40.4% in the root As content was reported in the As-40 treatment after foliar application of ICn-80 (Figure 2). Likewise, a significant reduction of 72.4% was monitored in the leaf As content in As-40 treatment supplemented with ICn-80.

### ICn improves nitrate reductase activity, carbohydrate content, and secondary metabolites in As-exposed peppermint

ICn proved promotive for the NR activity and significantly increased its activity by 13.9% over the control (Figure 3B). A maximum decrease of 12.6% in the NRA was reported in As-40 plants. A significant increase of 11.4% in As-40 treatment after ICn application was observed; therefore, the decrease in As-40 plants after ICn application compared to the DI water-sprayed control was only 1.1% (Figure 3B), signifying the mitigation of deleterious effects of As toxicity on the NRA. ICn-80 increased the activities of PAL and DXR significantly by 65.9 and 28.9%, respectively, over the DI water-treated plants. Lower levels of As (10 mg kg^−1^ soil) also increased the activities of the two enzymes; however, As-40 treatment recorded a significant decrease in the PAL activity by 12.8% over the control. Higher concentrations of As (20 and 40 mg kg^−1^ soil) led to a significant decrease in secondary metabolite accumulation. As-20 and As-40 treatments significantly decreased the total phenol content by 14.4 and 16%, respectively, over the control. Plants supplemented with ICn recorded a significant enrichment of 25.6% in the carbohydrate content compared to the control (Figure 3F). Due to As toxicity, the carbohydrate content was significantly reduced in As-40 treatment by 18.7% compared to the control. ICn-80 significantly alleviated the deleterious effect of As and augmented the carbohydrate content by 8.8% in As-40-treated plants (Figure 3F).

The adverse effect of As on the total phenol content was effectively mitigated by the application of ICn, and a significant increase of 17.3% was recorded in ICn-80 treatment as compared to the control (*P* < 0.05). A maximum content of total phenols was reported in the As-10+ICn-80 treatment (Figure 3D). A significant increase of 26.4% was reported in this treatment over the control (Figure 3D).

**Figure 4:**
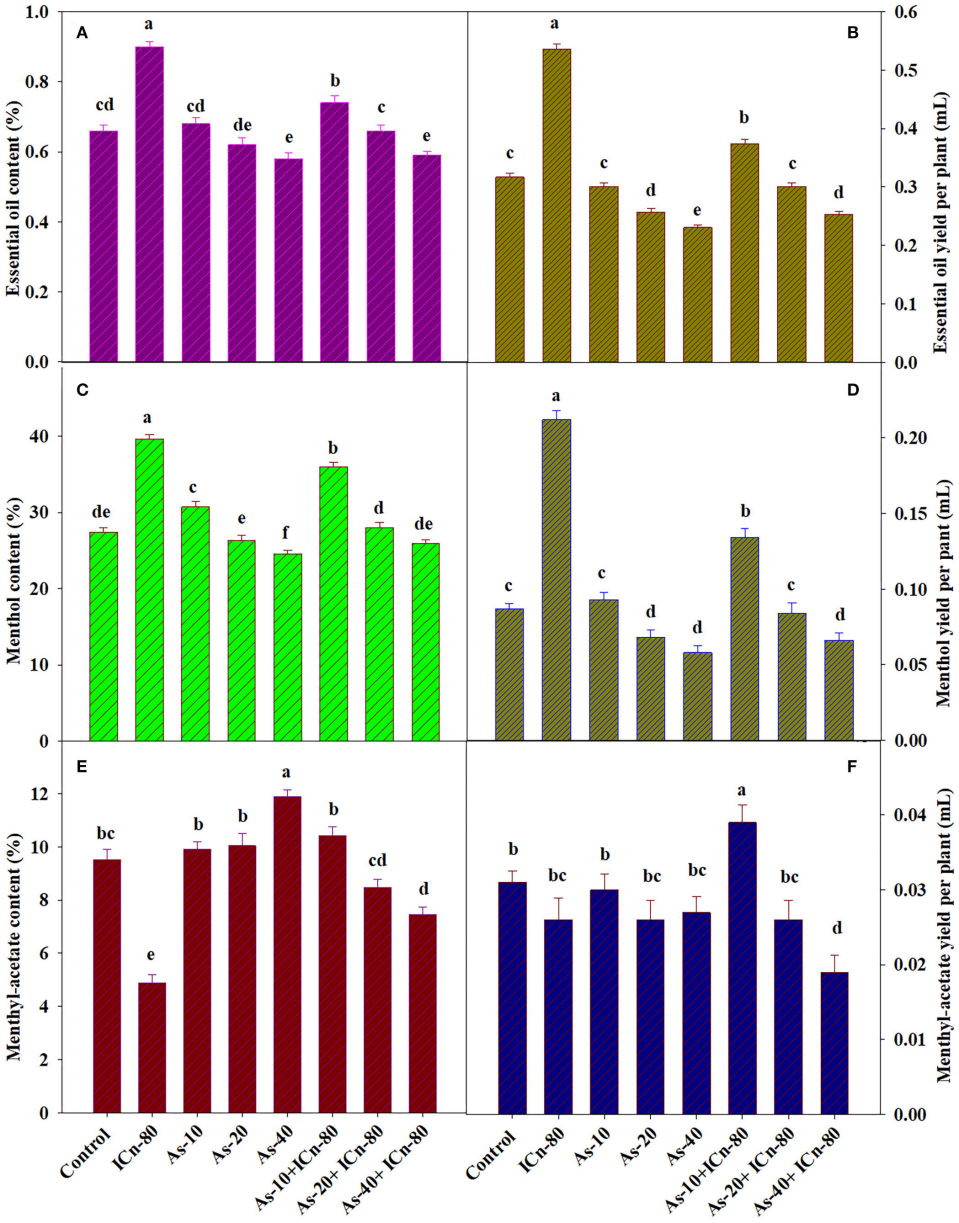
Essential oil content **(A)**, essential oil yield per plant **(B)**, menthol content **(C)**, menthol yield per plant **(D)**, menthyl acetate content **(E)**, and menthyl acetate yield per plant **(F)** of peppermint (*Mentha piperita* L.) at 150 days after transplanting with/without As stress (10, 20, or 40 mg kg^−1^ of soil, as Na_2_HAsO_4_·7H_2_O) and treated with ICn (80 mg L^−1^). Data are presented as treatment mean ± SE (*n* = 5). Bars with the same letters are not significantly different (*P* < 0.0).

The EO content and EO yield per plant were significantly enhanced by 36.4 and 69.1%, respectively, as compared to the DI water-sprayed control (*P* < 0.05; Figures 4A, B). The deleterious effect of As stress on the EO content and yield is clearly evident from the results. A significant reduction of 13.8% in the EO content was observed in As-40, while the yield of EO was significantly decreased by 37.2% over the control. The mitigatory effect of ICn is quite visible as the supplementation of 80 mg L^−1^ ICn on As-40 treatment augmented the content and yield of EO by 1.7 and 9.5%, respectively (Figures 4A, B).

**Figure 5:**
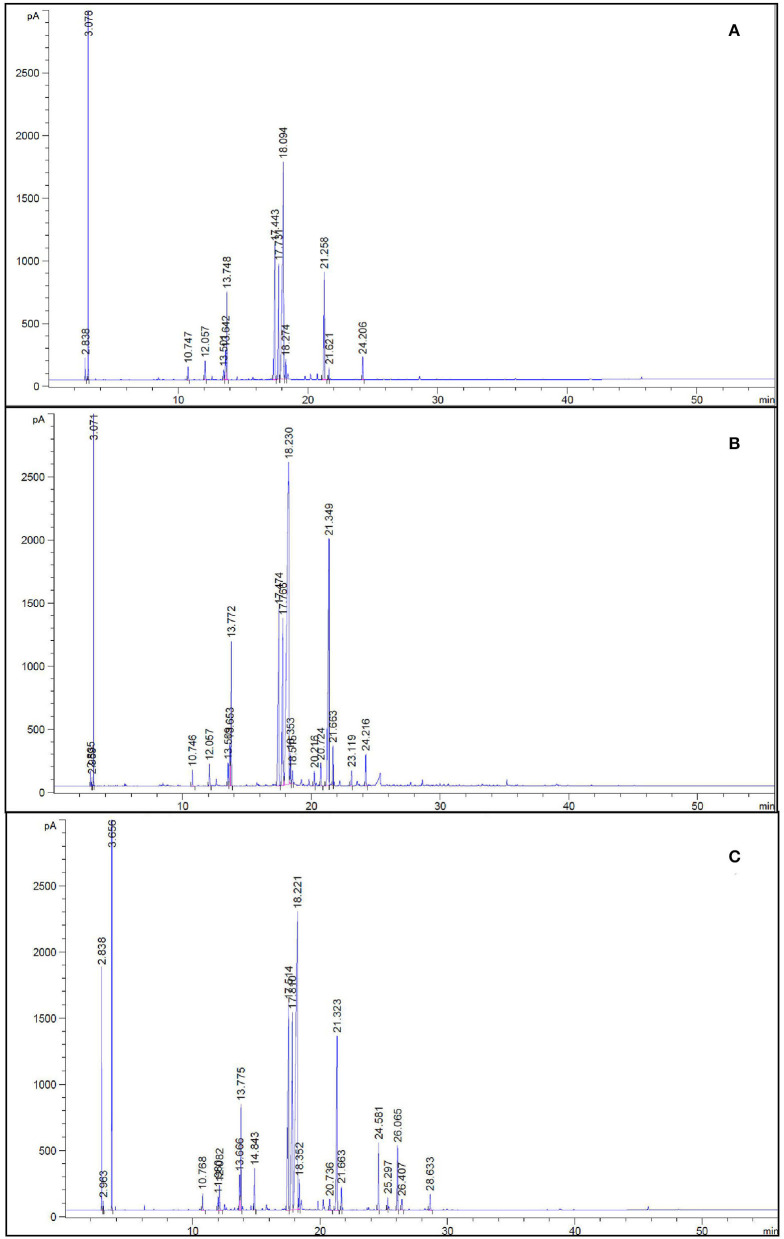
GC chromatograms of control (de-ionized water) **(A)**, ICn-80 **(B)**, and As-40+ICn **(C)** treatment plants displaying peaks of different active constituents of essential oil in peppermint (*Mentha piperita* L.) 150 days after transplanting with/without As stress (10, 20, or 40 mg kg^−1^ of soil, as Na_2_HAsO_4_·7H_2_O) and treated with ICn (80 mg L^−1^). Data are presented as treatment mean ± SE (*n* = 5).

The effect of ICn on the contents of menthol and menthyl acetate was opposite; while the content of menthol increased, the menthyl acetate content decreased significantly (*P* < 0.05; Figures 4C, E, 5). A significant increase of 44.4% over the control was recorded by ICn-80, while a significant decrease of 94.6% was observed in the content of menthyl acetate (Figure 5). As stress had a contradictory effect on the content of monoterpenes; the content of menthol was decreased maximally by 11.8% in As-40, while this treatment displayed the highest increase of 24.8% over the control in the menthyl acetate content (Figures 4C, E). The yield of menthol was observed to display highest values in ICn-80-treated plants with a significant increase of 143.6% over the control (Figure 4D), while highest menthyl acetate yield per plant was reported in As-10+ICn-80, with an increase of 25.8% over the DI water-sprayed plants (Figure 4F).

## Discussion

This study confirmed that As applied through soil severely affected the growth of plants. Among the three concentrations, As-40 was the most deleterious (Table 1). Increasing As concentration from 10 to 40 did not have a significant effect on the growth parameters studied. As toxicity adversely affects assimilation of carbon and nitrogen and impedes antioxidant activity, resulting in the decrease in different growth attributes, including plant biomass (Pandey and Gupta, 2015; Li et al., 2019; Nabi et al., 2021). Moreover, As stress impairs cellular functioning due to reaction with sulfhydryl groups of imperative enzymes and other cellular proteins, eventually leading to apoptosis (Pandey and Gupta, 2015; Li et al., 2019). The promotive effect of ICn on growth characteristics of peppermint in As-stressed peppermint can clearly be manifested in this study. The deleterious effects of differential As concentrations were considerably mitigated by the application of ICn (Table 1). Application of oligosaccharides manipulates vital plant metabolic events, improving their performance under normal and perturbed environmental circumstances (Ahmad et al., 2017; Sadiq et al., 2017; Singh et al., 2017; Nabi et al., 2019; Naeem et al., 2020). ICn enhances the growth in peppermint by recuperating plant metabolism through improved nutrient assimilation, photosynthesis, and enzyme activities (Ahmad et al., 2017; Jaleel et al., 2017; Ahmed et al., 2020).

The detrimental effects of As on peppermint photosynthesis were clearly observed (Figure 1). Diminution in the total leaf chlorophyll content due to As toxicity can be accredited to the impaired chlorophyll biosynthesis due to As-mediated impairment of a vital chlorophyll biosynthesis enzyme (α-aminolevulinic acid dehydratase) and/or due to ROS-induced breakdown of chlorophyll pigments and chloroplast membrane lipids (Kumar et al., 2014; Pandey and Gupta, 2015; Asgher et al., 2021; Zhang et al., 2021). As toxicity reportedly hinders the light harvesting apparatus, impairing the photosystem II activity, and in turn impedes the photosynthetic electron flow across the chloroplast thylakoids, thereby diminishing the assimilatory power (ATP and NADPH) producing potential which is fundamentally indispensable to fuel up the carbon fixation reactions (Duman et al., 2010; Nabi et al., 2019; Asgher et al., 2021; Lukacova et al., 2021). The potential energy deficit uncouples electron transport in the thylakoids, discontinuing the ATP synthesis possibly due to the exchange of inorganic phosphate by As in photophosphorylation (Singh et al., 2006; Duman et al., 2010; Nabi et al., 2021). RuBisCo activity was also severely affected by As toxicity (Figure 1). The large subunit of the enzyme, encoded by the plastid DNA, decreases in size under As toxicity (Ahsan et al., 2008, 2010). The decreased abundance of the enzyme signifies the possibility of As-induced interference of the gene expression of the chloroplast genome, as well as As-mediated impairment of the carbon fixation capacity (Abercrombie et al., 2008). ICn applied through leaves mitigated the As-induced damage on different photosynthetic attributes (Figure 1). ICn-induced improvement in NPR is possibly due to the improved PSII activity, which finds support from the increased chlorophyll fluorescence in contrast to the As-treated plants. Naeem et al. (2020) reported improved photosynthesis in As-treated *Artemisia* due to oligocarrageenan application. Augmentation in the values of different photosynthetic attributes in the current investigation indicates the improved CO_2_ fixation (increased chlorophyll content and CA and RuBisCo activities) and C assimilation (increased NPR, Fv/Fm, and *g*_*s*_), which consequently incorporates more carbohydrates (increased carbohydrate content; Figure 3) at the sink, leading to increased growth of the plant. Although the As-affected photosynthetic carbon metabolism is sparsely understood, it gives the impression that the metalloid decreases CO_2_ fixation, reducing the quantity of carbon accessible to the plant (Finnegan and Chen, 2012; Lukacova et al., 2021). Nitrogen (N) and C metabolism are interconnected in plants, and activity of N assimilating enzyme (NR) relies upon photosynthetic activity (Lillo et al., 2004). Under N deficiency, the chloroplast photosynthetic apparatus breaks down, leading to a sharp decline in NR activity (Davenport et al., 2015; Ahmad et al., 2019a). Significant diminution in the NR activity was witnessed in the As-stressed peppermint plants, and ICn-supplementation significantly increased the activity of the enzyme (Figure 3B). PSI-induced augmentation of photosynthetic electron flow is correlated with enhanced NR activity as this type of electron flow enhances the expression of NR genes (Sherameti et al., 2002). Improved NR activity in the existing study finds correlation with the ICn-mediated enhancement in the photosynthetic activity. Carbon input into metabolism is essentially significant for the plant, and As toxicity is known to impair the same, inhibiting the net photosynthesis (Nabi et al., 2019). Thus, improved values of photosynthetic parameters in As-treated plants are undoubtedly indicative of the role of ICn in improved fixation and assimilation of CO_2_, which increases the carbohydrate content in the plant and in turn alleviates the deleterious effects of As (Figure 3F). Plants supplemented with ICn recorded a significant enrichment in the carbohydrate content over the control (Figure 3F).

Among the different regulatory activities adopted by the plant under perturbed environmental conditions, regulation of antioxidant metabolism is of chief significance as it allows alleviation of membrane lipid peroxidation, which otherwise leads to severe injury to the plants (Imtiaz et al., 2015; Asgher et al., 2021). Amid the different species of As, As^V^ and As^III^ being redox-active metalloids, generate reactive oxygen species (ROS), which lead to membrane damage, redox imbalance, lipid peroxidation, and cytotoxicity (Srivastava et al., 2005; Finnegan and Chen, 2012; Nabi et al., 2021; Zhang et al., 2021). The worst outcome of ROS is the peroxidation of cells and organelle membrane lipids, which enhances electrolyte leakage and disrupts metabolic pathways like photosynthesis and respiration by disintegrating the electron transport system of semiautonomous organelles (mitochondria and chloroplast) (Lukacova et al., 2021; Nabi et al., 2021). As toxicity-induced ROS disrupts the chloroplast membrane structure and consequently breaks down the photosynthetic machinery (thylakoids), reducing the light-absorbing pigments and thereby lowering the photosynthetic rate (Finnegan and Chen, 2012; Asgher et al., 2021; Nabi et al., 2021; Zhang et al., 2021). The estimation of thiobarbituric acid-reactive substances (TBARSs) is an important tool to quantify the electrolyte leakage (Shri et al., 2009). The study witnessed a significant enhancement in TBARSs in plants exposed to As stress (Table 2). Similar to TBARSs, the H_2_O_2_ content was enhanced in plants exposed to As stress (Figure 3). The elicitor effect of ICn becomes clearly evident from the significant reduction in the TBARS and H_2_O_2_ content after supplementation of ICn to As-exposed plants (Table 2). ROS scavenging is vital in metal/metalloid-stressed plants for efficient metabolism, and a variety of antioxidants (enzymatic and non-enzymatic) and osmolytes exist in plants to serve the purpose (Shri et al., 2009). Amid these, SOD constitutes the primary line of defense and facilitates scavenging of active oxygen-free radicals to H_2_O_2_, while POX-like enzymes help in conversion of H_2_O_2_ to H_2_O (Shri et al., 2009). SOD, POX, and APX activities were upregulated in As-stressed plants in the current study (Table 2). Furthermore, the content of proline, a low-molecular weight highly soluble and compatible osmolyte, was significantly increased in As-exposed peppermint supplemented with ICn. ICn alone, or combined with As, significantly augmented the proline content in peppermint. Proline facilitates cellular osmotic adjustment, stabilizes enzymes and proteins, and maintains membrane integrity under high ROS accumulation (Hayat et al., 2012). Likewise, the activity of GR in As-treated plants was considerably higher than that in control and ICn-80 treatments (Table 2). A reduced form of glutathione (GSH), produced by glutathione reductase (GR) in plants, comprises an indispensable constituent of the ascorbate–glutathione cycle, which is obligatory to combat the oxidative damage in plants (Kaya et al., 2020).

As-induced ROS production, reduction in the growth, and impaired metabolic activities in plants become more severe upon As accumulation (Kumar et al., 2015; Yang et al., 2021). Significant accumulation of As in the root and leaf tissues in the peppermint plants exposed to differential As concentrations with a maximum accumulation in As-40 treatment was observed (Figure 2). Application of ICn *via* leaves significantly decreases the accretion of As in peppermint roots and leaves. ICn-induced mitigation of As toxicity is endorsed to reduced root and leaf As accretion. Furthermore, ICn reduced the root-to-shoot translocation of As in peppermint, which is imperative for the regulation of As-affected photosynthesis, as well as antioxidative metabolism (Figure 2). Reduction in As accumulation and root-to-shoot translocation can be attributed to the downregulation of As transporters in the roots and those involved in translocation to the shoot (Naeem et al., 2020). Moreover, increased peroxidase activity is reported to minimize As accumulation by facilitating the apoplastic lignification (Kidwai et al., 2019).

**Figure 6:**
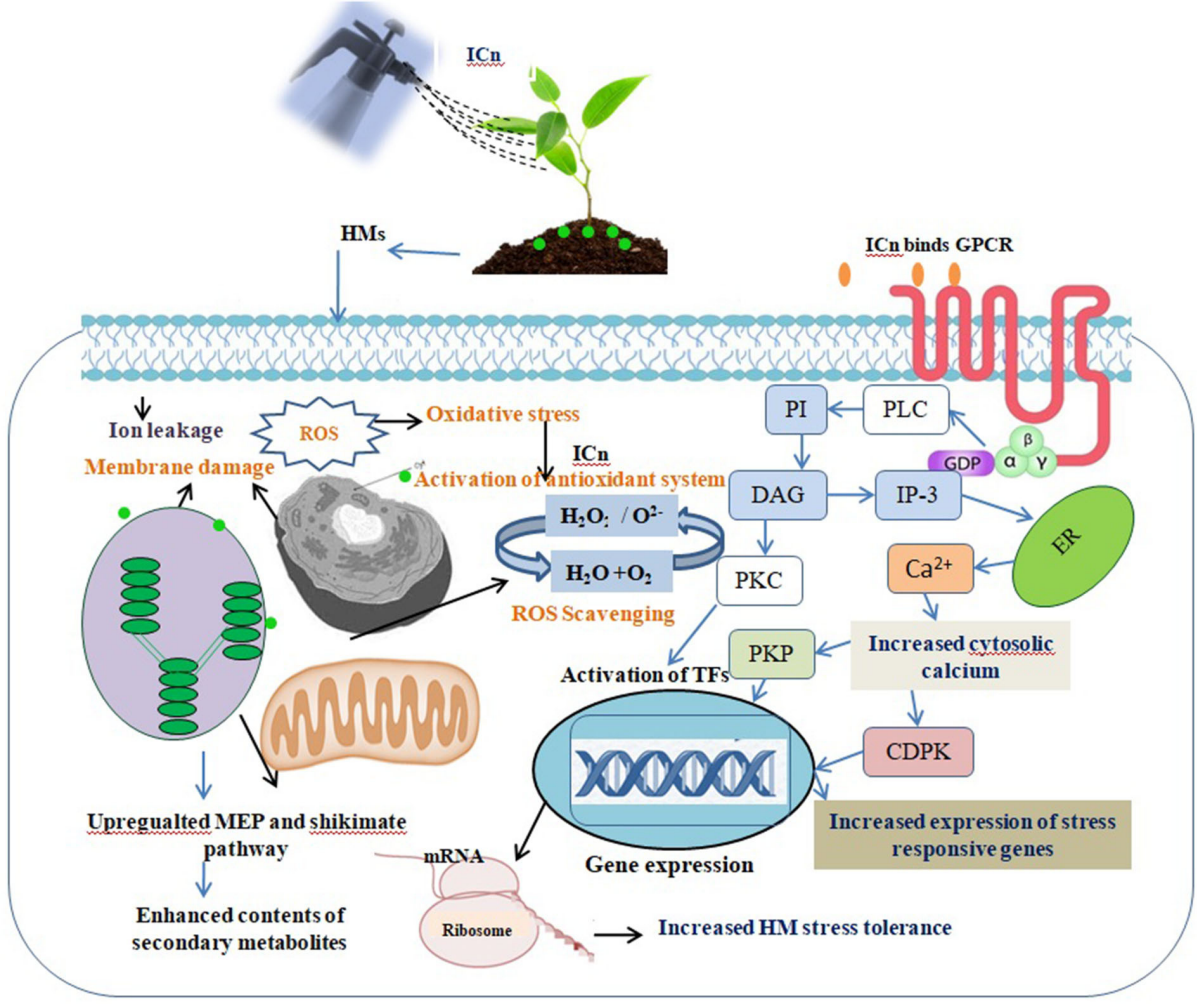
Irradiated chitosan (ICn) binds to the plasma membrane receptors transmitting the signal through protein kinase (PK). ICn detoxifies the disproportionate ROS produced due to arsenic (As) stress by increasing the expression of antioxidative enzymes. Activation of phospholipase C (PLC) and G-protein signaling pathways enhances the concentration of cytosolic Ca^2+^, which, consequently, activates cell-dependent protein kinase (CDPK) and protein kinase phosphatase (PKP). These activated proteins stimulate various transcription factors, which in turn stimulates the expression of various gene response elements, leading to stimulation of different secondary metabolism pathways in As-stressed peppermint [19, 27, 94].

EO accumulates inside the secretory cell leucoplasts of the peltate glandular trichomes (PGTs) *via* two different pathways, namely, methyl erythritol phosphate (MEP) and mevalonic acid pathways, in the chloroplast and cytoplasm, respectively (Gershenzon et al., 2000; Zhao et al., 2013; Johnson et al., 2017). Activities of two vital regulatory enzymes of secondary metabolite biosynthesis, one regulating the biosynthesis of phenols and the other regulating the EOs (terpenes), were assessed to gain understanding of the relationship between As stress and secondary product accumulation in peppermint. PAL and DXR are shikimic acid pathway and MEP pathway enzymes, respectively (Ahmad et al., 2019a; Ahmed et al., 2020). Higher As concentrations (20 and 40 mg Kg^−1^ soil) significantly decreased the secondary metabolite accumulation (EOs and phenols) (Figures 4, 5). The decrease in the contents of EOs and phenols is endorsed to the decreased activities of PAL and DXR in As-stressed plants, as well as the severely affected photosynthesis in peppermint. Foliar application of ICn enhanced the activities of PAL and DXR, leading to the enhanced biosynthesis of the EOs and phenols in As-exposed plants (Figure 6). The adverse effect of As on phenol accretion was significantly mitigated by ICn supplementation (Figure 3D). Likewise, the deleterious effect of As stress on the EO content and EO yield per plant is apparent from the results. EO production was significantly enriched by ICn application in peppermint as compared to the DI water-sprayed control (Figures 4A, B). The alleviatory effect of ICn is apparent as the application of ICn-80 on As-40 plants augmented the content and yield of EO (Figures 4A, B). ICn-mediated enhancement in the values of photosynthetic attributes (CA activity, NPR, g_s_, Fv/Fm, and RuBisCo) augments more carbohydrates, which improves the carbohydrate content of the plant (Figure 3F). Carbohydrates, synthesized in chloroplasts and catabolized in the cytoplasm and mitochondria in plants, are diverted to secondary metabolite biosynthesis (phenols and EOs) (Singh et al., 1990, 2006). As disrupts C assimilation, which in turn affects the potential diversion of stored carbohydrates to secondary metabolism (Duman et al., 2010; Nabi et al., 2019). As reflected from the terpene accumulation pattern, EO accretion is predominantly governed by the quantity of photosynthates switched to secondary product biosynthesis (Gershenzon et al., 2000; Zhao et al., 2013). Likewise, enhanced accretion of phenols in response to ICn is accredited to increased PAL activity. Increased phenol content due to oligosaccharide application corroborates previous reports (Swamy and Rao, 2009; Dar et al., 2015; Ahmad et al., 2017, 2019b). Similarly, over-expression of DXR in transgenic peppermint has been reported to enhance the EO biosynthesis and accumulation (Croteau et al., 2005). Increased EO content and yield due to ICn application are accredited to the increased DXR activity and photosynthesis in the current study. As stress decreases the carbohydrate content, photosynthesis, activities of DXR, and other enzymes (RuBisCo, NR, CA), affecting the accumulation of EO severely. Exposure of *Artemisia* to As is reported to reduce the content of artemisinin (Naeem et al., 2020). ICn enriches the content and yield of EO by increasing the activity of DXR, as well as improves photosynthesis in plants (Ahmad et al., 2017; Jaleel et al., 2017; Ahmed et al., 2020).

The effect of ICn on the contents of monoterpenes in the EO, menthol, and menthyl acetate was contrasting, whereas the menthol content increased and the menthyl acetate content decreased significantly (Figures 4C,E, 5). Likewise, As stress had a contradictory effect on the content of monoterpenes; the content of menthol was decreased, while that of menthyl acetate increased significantly over the control (Figures 4C,E). Highest values for the yield of menthol were reported in ICn-80 treatment (Figure 4D), while maximum menthyl acetate yield per plant was reported in As-10+ICn-80 treatment (Figure 4F). ICn increases the content of menthol probably by upregulating the reductase enzyme involved in its biosynthesis (Ahmad et al., 2017, 2019b). As-induced reduction in the menthol content is endorsed to decreased photosynthesis and downregulation of imperative reductase enzyme involved in menthol biosynthesis (Nabi et al., 2019). As revealed from the biosynthetic pathway of terpenes in peppermint, the content of menthol increases at the cost of menthyl acetate, and vice versa (Ahmad et al., 2017). A decreased content of menthol has been observed to be related with the enhancement in the menthyl acetate content (Ahmad et al., 2018). Conclusively, the current study elucidates that ICn serves as the elicitor and improves the overall performance of peppermint exposed to As toxicity (Figure 6).

## Conclusion

This study elucidates that foliar application of ICn alleviates the disadvantageous effects of As by reducing the absorption of As, decreasing its root-to shoot translocation, and fortifying the antioxidative metabolism (enzymatic and non-enzymatic). Moreover, improved photosynthesis and nutrient assimilation due to enhanced activities of imperative primary and secondary metabolic enzymes (RuBisCo, CA, NR, PAL, DXR) are endorsed to ICn application, which leads to increased accretion of different secondary metabolites (EOs and phenols). The secondary products, particularly phenols, play a vital role in combating As-induced toxicity, in addition to their chemo-ecological functions in plants. Consequently, it is concluded that ICn applied through foliage at a concentration of 80 mg/L can prove as an efficient growth promoter for economically vital crop plants like peppermint and can help in eliminating metal/metalloid-induced toxicity, thereby helping in obtaining higher yields in EO-containing crops grown in As-contaminated soils.

## Data availability statement

The raw data supporting the conclusions of this article will be made available by the authors, without undue reservation.

## Author contributions

BA, PA, and JR involved in conceptualization. TD, MK, and AA designed the experimental setup. BA, TD, and MK performed the experiment and wrote the manuscript. BA, JR, and YC helped with writing. PA, JR, and YC revised/edited the manuscript. BA, AA, and TD performed the statistical analysis. All authors read and approved the final manuscript.

## Conflict of interest

The authors declare that the research was conducted in the absence of any commercial or financial relationships that could be construed as a potential conflict of interest.

## Publisher's note

All claims expressed in this article are solely those of the authors and do not necessarily represent those of their affiliated organizations, or those of the publisher, the editors and the reviewers. Any product that may be evaluated in this article, or claim that may be made by its manufacturer, is not guaranteed or endorsed by the publisher.
